# Effects of Biomechanical Testing Using a Synthetic Ligament Fabricated from Polyhydroxyalkanoate Biopolyesters for Lateral Ulnar Collateral Ligament Reconstruction in Cadaver

**DOI:** 10.3390/polym18040514

**Published:** 2026-02-19

**Authors:** Anucha Wimoonchart, Tulyapruek Tawonsawatruk, Anuchan Panaksri, Nuttapol Tanadchangsaeng

**Affiliations:** 1 College of Biomedical Engineering, Rangsit University, Pathumthani 12000, Thailand; 2 Department of Orthopaedics, Faculty of Medicine, Ramathibodi Hospital, Mahidol University, Bangkok 10400, Thailand

**Keywords:** synthetic ligament, polyhydroxyalkanoates, lateral ulnar collateral ligament, cadaver

## Abstract

An injury to the elbow’s lateral ulnar collateral ligament (LUCL) is an orthopedic emergency that can impair joint stability and functional biomechanics throughout the upper extremity. The development and application of synthetic ligament substitutes, particularly short-chain-length and medium-chain-length polyhydroxyalkanoate (SCL-PHA and MCL-PHA) co-polymers, represent a promising innovation for lateral elbow stabilization. This experimental cadaveric study aimed to (1) compare biomechanical parameters of torque and angular rotation among control, damage, repair, and reconstruction groups and (2) compare stress and strain responses across the same groups. Twenty-four cadaveric elbows were allocated among six experimental conditions. The control group consisted of intact elbows (*n* = 4), while the damage group (*n* = 4) involved transection of the anterior capsule and extensor carpi radialis brevis (ECRB) to simulate ligament injury. The repair group (*n* = 4) underwent anterior capsular suturing. The reconstruction group (*n* = 12) was divided into three subgroups: palmaris longus (PL) autograft alone, PL with SCL-PHA co-polymer augmentation, and PL with MCL-PHA augmentation. Biomechanical testing measured maximum torque, angular displacement, shear stress, and strain, with statistical analysis conducted using descriptive statistics, one-way ANOVA, and post hoc multiple comparisons. The results demonstrated that maximum torque (F = 24.930, *p* < 0.001) and maximum shear stress (F = 8.130, *p* < 0.001) significantly differed among groups. The control group exhibited the highest mechanical performance (30.700 ± 9.368 Nm and 0.880 ± 0.216 MPa), whereas the damage group showed the lowest values (10.300 ± 2.904 Nm and 0.210 ± 0.073 MPa). The reconstruction group using palmaris longus with SCL-PHA co-polymer reinforcement (RC-PLSCL) demonstrated torque (29.550 ± 7.656 Nm) and shear stress (0.610 ± 0.206 MPa) comparable to those of the control group (*p* > 0.05), indicating near-physiological mechanical behavior. These findings suggest that SCL-PHA co-polymer augmentation offers superior biomechanical restoration relative to standard repair and other reconstruction strategies, highlighting its potential as an advanced biomaterial for ligament reconstruction in LUCL injuries.

## 1. Introduction

Injury to the lateral ulnar collateral ligament (LUCL) of the elbow is a primary cause of posterolateral rotatory instability (PLRI), a pathological condition that compromises the joint’s structural stability and functional kinematics [1,2]. LUCL injuries are commonly attributed to traumatic events, repetitive valgus loading, or iatrogenic causes during lateral elbow procedures [3,4]. Conventional surgical management typically involves either direct ligament repair or reconstruction using autografts such as the palmaris longus tendon [5,6]. However, these approaches often yield inconsistent outcomes, primarily due to limitations in graft mechanical strength, donor site morbidity, and the inability to fully restore the native biomechanical properties of the ligament [7,8]. Consequently, there has been increasing interest in the development of synthetic and bio-derived ligament substitutes that can more accurately replicate the viscoelastic and structural characteristics of natural ligaments [9].

Medium-chain-length polyhydroxyalkanoate (MCL-PHA) is a biodegradable and biocompatible polyester exhibiting hyper-elastic mechanical behavior, with flexibility comparable to that of human tendon tissue [10]. These favorable characteristics have led to its consideration as a promising material for artificial tendon or ligament implants in orthopedic and reconstructive surgery [11,12]. In a recent investigation, Tawonsawatruk et al. [13] developed an artificial tendon composite derived from MCL-PHA fabricated via a solution-casting technique, forming cylindrical and rectangular constructs with predesigned perforations that allowed integration of torn human tendon fragments to create a composite tendon graft. Mechanical testing revealed that the cylindrical MCL-PHA configuration, threaded in a zigzag pattern through three perforations, achieved the highest maximum tensile strength of 56 MPa, closely approximating the ultimate tensile stress of native human tendon (50–100 MPa) [13]. Moreover, in vitro experiments using patient-derived fibroblast cells demonstrated strong cellular adhesion and proliferation on the MCL-PHA surface, accompanied by high viability and robust collagen synthesis, promoting the chondrogenic induction of tendon cells [13]. Complementary in vivo biocompatibility tests performed in a rat subcutaneous implantation model further showed that MCL-PHA exhibited excellent tissue compatibility, comparable to medical-grade silicone, without eliciting inflammatory or adverse host responses [13]. Building upon these findings, the present study employed MCL-PHA materials developed by Tawonsawatruk et al. [13] as the principal biomaterial for biomechanical testing in cadaveric specimens, aiming to evaluate their feasibility as synthetic grafts for LUCL reconstruction. This research represents a translational step from laboratory-based biomaterial development toward practical surgical implementation, offering biomechanical perspectives under near-physiological loading conditions. The findings are expected to expand the potential clinical applications of PHA-based composite materials in ligament reconstruction and bioengineered tendon–ligament interfaces.

Cadaveric biomechanical testing serves as a critical platform for evaluating the functional performance of ligament repair and reconstruction techniques under controlled, near-physiological conditions [14,15]. Previous cadaveric investigations have explored various reconstruction strategies, including tendon grafting, internal brace augmentation, and minimally invasive reconstruction using fascia or tendon patches. Each of these approaches has demonstrated significant improvements in torque resistance and rotational stability compared to injured or unrepaired states [15,16]. For example, Shukla et al. [15] showed that the posterior bundle of the medial ulnar collateral ligament (MUCL) helps keep the elbow from dislocating when it is under valgus stress. Scheiderer et al. [16] showed that internal bracing of the LUCL brought posterolateral rotatory instability (PLRI) back to levels that were almost normal. Despite these advancements, there remains a paucity of comparative data assessing the biomechanical efficiency of PHA-based synthetic ligaments relative to native, damaged, and repaired tissues, particularly within the context of LUCL reconstruction. Addressing this knowledge gap is essential for validating the translational potential of PHA-based biomaterials in restoring physiological joint stability and for advancing bioengineered ligament replacement strategies.

Consequently, the current study’s objective was to assess the biomechanical properties of synthetic ligaments made from PHA in a cadaveric model of lateral elbow reconstruction. The investigation specifically compared peak torque, torque angle, peak shear stress, and shear strain among four experimental groups: control, damage, repair, and reconstruction (RC-PL, RC-PLSCL, and RC-PLMCL). The findings of this study are expected to provide foundational evidence supporting the use of PHA-based materials in ligament reconstruction and to advance the development of bioengineered ligament substitutes that more closely emulate native biomechanical function.

## 2. Materials and Methods

### 2.1. Materials

The materials used in this study consisted of embalmed cadaveric upper limbs and synthetic ligament constructs. Twenty-four (24) upper limbs from human cadaveric donors were obtained from the Anatomy Laboratory, Faculty of Science, Rangsit University, Thailand. The specimens were categorized into four groups: the control group (*n* = 4 arms) representing intact ligaments, the damage group (*n* = 4 arms) representing mechanically injured ligaments, the repair group (*n* = 4 arms) representing surgically repaired ligaments, and the reconstruction group (*n* = 12 arms), which was further subdivided into three subgroups (RC-PL, RC-PLSCL, and RC-PLMCL, four arms each) based on the type of material used for ligament reconstruction. All specimens were selected by applying predefined inclusion criteria, namely, intact elbow anatomy, donor age ranging from 60 to 80 years, absence of any history of elbow-related trauma or surgical intervention, and preservation for a minimum of one year using a uniform embalming protocol. All cadavers were legally donated for research and educational purposes, and the study protocol was reviewed and approved by the Institutional Ethics Committee for Human Research, Rangsit University.

The synthetic ligament materials used in the reconstruction groups included medium-chain-length polyhydroxyalkanoate (MCL-PHA) and short-chain-length polyhydroxyalkanoate (SCL-PHA) composites, developed based on the study by Tawonsawatruk et al. [8], which demonstrated suitable biomechanical strength, elasticity, and biocompatibility for tendon and ligament reconstruction.

Additional materials and instruments were used to support the biomechanical testing and sample preparation. The primary testing apparatus included a universal testing machine (LR-10K, LLOYD Instruments Ltd., Fareham, UK) for torque and shear evaluation, a digital thickness gauge (Mitutoyo Corp., Kawasaki, Japan), a coarse balance (OHAUS Corp., Parsippany, NJ, USA), and a precision analytical balance (Sartorius AG, Göttingen, Germany), a laboratory centrifuge (Eppendorf AG, Hamburg, Germany), a laboratory heat box/oven (Memmert GmbH + Co. KG, Schwabach, Germany), and a vortex mixer (IKA Works GmbH & Co. KG, Staufen, Germany). Bone tunnel preparation was performed using a standard electric bone drill (R6002 FIXMAN Pro Power Screwdriver, Ningbo Jiejie Tools Co., Ltd., Ningbo, China). Ligament fixation was achieved using orthopedic suture anchors (Mini-Revo suture anchor, CONMED Corp., Largo, FL, USA), together with standard measuring tools. Laboratory accessories consisted of custom-designed molds, Parafilm, syringes, sandpaper, and surgical forceps. All reagents used in this study were of analytical grade, including distilled water, chloroform, 75% glycerine, methanol, and deionized (DI) water. These materials and instruments ensured precision, reproducibility, and control in evaluating the biomechanical behavior of both biological and synthetic ligament specimens under standardized conditions.

### 2.2. Experimental Procedures

This experimental study employed two types of polyhydroxyalkanoates (PHAs): a short-chain-length PHA co-polymer (SCL-PHA co-polymer, specifically poly(3-hydroxybutyrate-co-3-hydroxyhexanoate) [PHBHHx]; purchased from Bluepha Co,. Ltd., Jiangsu, China) and a medium-chain-length PHA (MCL-PHA) that was produced and fabricated in the laboratory at College of Biomadical Engineering, Rangsit University [13]. These biomaterials were used to fabricate synthetic ligaments, which were subsequently subjected to biomechanical testing using preserved cadaveric specimens in which the lateral elbow ligaments had been surgically reconstructed.

#### 2.2.1. Fabrication of Synthetic Ligaments

SCL-PHA co-polymer and MCL-PHA were separated based on their differential solubility in methanol–chloroform mixtures to obtain two polymer fractions with distinct physical characteristics. For scaffold fabrication, each polymer type was dissolved in chloroform to prepare a polymer solution with a defined concentration. Specifically, MCL-PHA was dissolved to obtain an 8% (*v*/*v*) solution with a total volume of 20 mL, which was then cast into a cylindrical mold measuring 2 mm in diameter and 150 mm in length. The solution was allowed to evaporate under controlled laboratory conditions at room temperature for 24–48 h without heat application to ensure uniform solvent removal. A similar solution-casting technique was applied for SCL-PHA co-polymer using an equivalent polymer-to-solvent ratio, producing scaffolds of the same dimensions. The resulting cylindrical scaffolds were engineered to replicate the geometry and tensile characteristics of natural ligament fibers. All fabricated ligaments were subsequently removed from the molds, conditioned, and sterilized prior to their use in ligament reconstruction procedures.

#### 2.2.2. Characterization of PHA Materials

The polyhydroxyalkanoate (PHA) materials employed for ligament augmentation in this study comprised medium-chain-length PHA (MCL-PHA) and a short-chain-length PHA co-polymer (SCL-PHA; poly(3-hydroxybutyrate-co-3-hydroxyhexanoate), PHBHHx). These two PHA systems were selected to represent distinct structure–property profiles relevant to load-bearing soft-tissue reconstruction, particularly differences in crystallinity, chain mobility, and elastomeric behavior.

Material characterization was conducted based on previously established and comprehensive investigations by Tawonsawatruk et al. [13], together with supporting polymer biomaterials literature [14], under fabrication protocols identical to those used in the present study. The same polymer sources, solvent-casting procedures, scaffold geometry, and post-fabrication conditioning were applied to ensure material consistency and reproducibility across studies. Accordingly, the reported physicochemical and mechanical properties are considered representative baseline characteristics of the PHA scaffolds used in the current cadaveric experiments.

Molecular-weight characteristics of PHBHHx assessed by gel permeation chromatography (GPC) in representative systems have demonstrated weight-average molecular weights on the order of 10^5^ g/mol, indicating sufficient chain entanglement to support mechanical integrity under tensile loading. Thermal behavior evaluated by differential scanning calorimetry (DSC) has shown that SCL-PHA co-polymers exhibit relatively higher crystallinity, corresponding to increased stiffness and tensile resistance, whereas MCL-PHA displays a semi-crystalline structure with enhanced chain mobility, conferring pronounced elastomeric and hyperelastic behavior.

Surface morphology characterized by scanning electron microscopy (SEM) in the referenced studies revealed smooth and continuous scaffold surfaces without observable microcracks or gross structural defects, both at the surface and across cross-sections. Such morphological features are indicative of homogeneous polymer distribution and mechanical robustness and have been associated with favorable material–tissue interactions in prior biological evaluations [13,14]. In the present study, identical fabrication and conditioning procedures were applied, minimizing variability attributable to scaffold morphology.

Baseline mechanical properties of the PHA scaffolds evaluated independently of ligament reconstruction have been previously reported [13,14]. Tensile testing demonstrated that MCL-PHA exhibits hyperelastic behavior with high elongation at break, while ultimate tensile strength is dependent on construct configuration and geometry. Notably, cylindrical MCL-PHA-based composite constructs achieved an ultimate tensile strength of approximately 56 MPa, approaching the reported tensile strength range of native human tendon. In contrast, SCL-PHA co-polymers demonstrated higher tensile strength and stiffness with reduced extensibility, consistent with their higher crystalline content.

Regarding stability during biomechanical testing, PHAs are known to exhibit high short-term physicochemical stability under dry and quasi-static testing conditions. No degradation, swelling, or softening of the polymer scaffolds was observed during the duration of specimen preparation or mechanical testing in the present study. Prior to cadaveric implantation, all PHA constructs were sterilized using a validated low-temperature protocol consisting of ethanol immersion, phosphate-buffered saline rinsing, laminar-flow drying, and ultraviolet exposure, which has been shown to preserve surface morphology, mechanical integrity, and biocompatibility [13].

Collectively, these molecular, thermal, morphological, mechanical, and stability characteristics provide a material-science framework for interpreting the biomechanical performance of PHA-augmented LUCL reconstruction in the present cadaveric model, while acknowledging that scaffold-level properties were derived from rigorously characterized materials fabricated under identical conditions.

#### 2.2.3. Biomechanical Testing on Cadaveric Specimens

**Figure 1:**
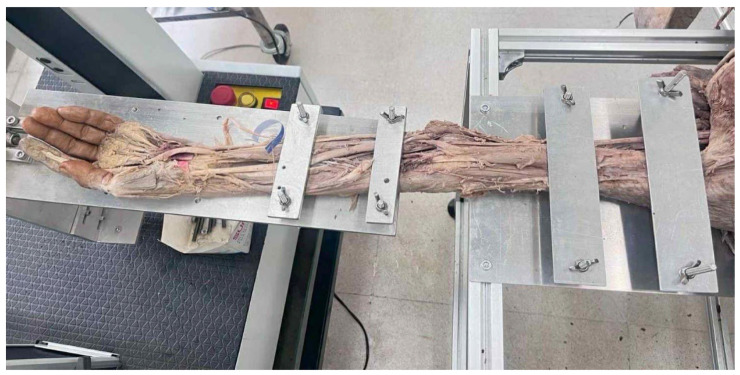
The image depicts an arm with intact muscles and ligaments associated with the elbow.

Biomechanical testing was conducted using preserved cadaveric upper limbs to evaluate the mechanical behavior of the lateral elbow ligaments under controlled loading conditions. All specimens were tested using a LLOYD Instruments LR-10K universal testing machine, capable of measuring torque, shear stress, and angular deformation during rotational loading. Each arm was securely mounted in a custom-designed fixture with the humerus rigidly fixed and the forearm aligned along the loading axis (Figure 1).

External rotation was applied to the forearm using a linear actuator at a constant displacement rate of 100 mm/min. Given an effective lever arm length of approximately 100 mm from the elbow rotation center to the point of load application, this displacement rate corresponds to an angular rotation rate of approximately 1°/s. This loading protocol represents a quasi-static testing condition, selected to minimize rate-dependent viscoelastic effects and to allow reliable assessment of structural stiffness, torque resistance, and failure characteristics of the ligament constructs, rather than to simulate high-velocity injury mechanisms. Rotational loading was continued until complete elbow dislocation occurred, at which point the measured torque dropped to zero [17].

The control group (*n* = 4) consisted of intact elbows with preserved musculature and ligaments. These served as the reference standard for native biomechanical properties, providing baseline measurements for maximum torque, angular displacement, and shear stress.

**Figure 2:**
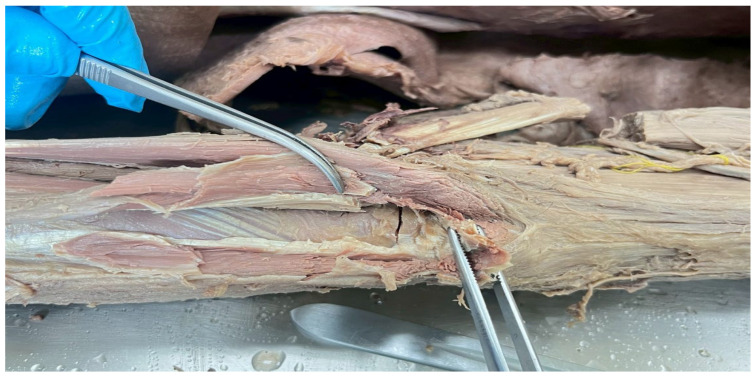
The image displays a cut of the anterior capsule, the extensor carpi radialis brevis muscle, and LUCL.

The damage group (*n* = 4) was used to simulate ligamentous instability by complete transection of the lateral ulnar collateral ligament (LUCL) at its humeral origin, together with transection of the anterior capsule and the extensor carpi radialis brevis (ECRB) muscle, thereby disrupting the primary lateral stabilizing structures of the elbow and inducing a posterolateral rotatory instability-like condition. This procedure resulted in a measurable reduction in torque resistance and rotational stability, reflecting the biomechanical consequences of LUCL deficiency, as shown in Figure 2.

**Figure 3:**
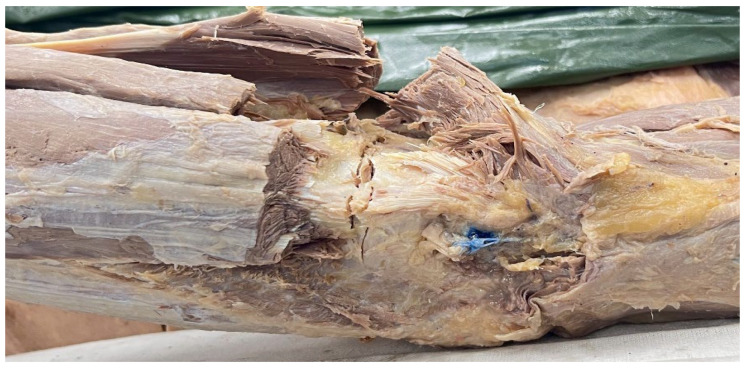
LUCL repair procedure.

In the repair group (*n* = 4), the anconeus muscle, ECRB, anterior capsule, and LUCL were transected at their origin to simulate a complete ligament rupture. The LUCL was then repaired using a FiberTape Mini-Revo system following the surgical technique [17], in which a 2.7 mm hole was drilled at the lateral epicondyle to anchor the graft [17]. The ligament was tensioned at 90° of elbow flexion prior to fixation to ensure appropriate anatomical alignment. This method aimed to restore the continuity of the LUCL and improve resistance to external rotation during mechanical testing (S1), as shown in Figure 3.

**Figure 4:**
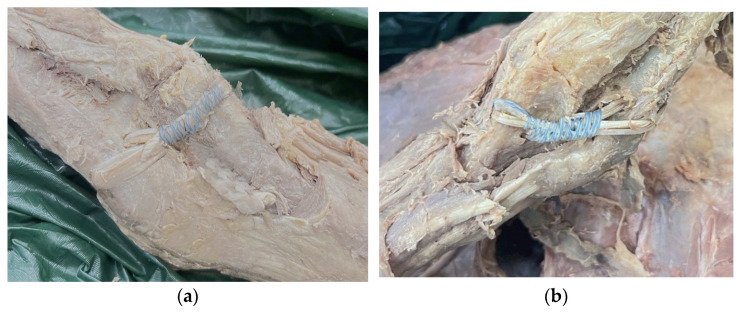
(**a**) Demonstration of repair using the palmaris longus tendon and SCL-PHA. (**b**) Demonstration of ligament reconstruction using palmaris longus tendon graft combined with medium-chain-length polyhydroxyalkanoates (MCL-PHAs).

For the reconstruction group (*n* = 12), all specimens underwent the same initial ligament and muscle resection as in the repair group. Four bone tunnels (3.2 mm in diameter) were created—two in the ulna and two in the humerus—to accommodate graft fixation [18]. Three reconstruction subgroups were established based on the graft material used: (1) RC-PL, reconstructed using a 15 cm palmaris longus tendon (S2); (2) RC-PLSCL, reconstructed using a palmaris longus tendon combined with short-chain-length polyhydroxyalkanoate (SCL-PHA co-polymer); and (3) RC-PLMCL, reconstructed using a palmaris longus tendon combined with medium-chain-length polyhydroxyalkanoate (MCL-PHA). All grafts were sutured using 2-0 surgical thread and secured with the FiberTape Mini-Revo docking technique [19] while maintaining 90° elbow flexion during fixation (S3), as shown in Figure 4.

After each reconstruction, specimens were mounted on the testing machine, and external rotational loading was applied at the same rate as in the other groups (S4). Measurements of peak torque, torque angle, peak shear stress, and shear strain were recorded to compare mechanical performance across all conditions—control, damage, repair, and reconstruction. This experimental setup enabled systematic evaluation of how PHA-based synthetic ligament constructs influenced the restoration of elbow joint stability under near-physiological loading conditions.

### 2.3. Statistical Analysis

Descriptive statistics were used to summarize the biomechanical variables and are reported as mean ± standard deviation for each experimental group. Data normality was assessed using the Shapiro–Wilk test, which indicated no significant deviation from a normal distribution (*p* > 0.05). Accordingly, one-way analysis of variance (ANOVA) was applied to compare torque, angular displacement, shear stress, and shear strain among the experimental groups. When statistically significant differences were identified, post hoc multiple comparisons were performed to determine specific intergroup differences. A significance level of α = 0.05 was adopted for all statistical analyses.

## 3. Results

### 3.1. Control Group Analysis

Biomechanical analysis of the control group (CT01–CT04) revealed a mean peak torque of 30.07 ± 9.368 Nm, with a mean maximum angular displacement of 245.5 ± 44.814°. CT02 demonstrated the highest peak torque (38.64 Nm), whereas CT04 showed the lowest (20.08 Nm), indicating moderate variability in torsional strength among specimens. Torque increased progressively with angular displacement, peaking at approximately 250–270° before declining, corresponding to the onset of irreversible elbow joint deformation.

The torque–angle relationship exhibited a nonlinear pattern, with a gradual torque increase at small angle range (0–100°), followed by a rapid rise between 100 and 250°, and plateauing or declining beyond 270°. This pattern reflects the characteristic mechanical response of ligament tissue under progressive tensile loading approaching joint dislocation.

**Figure 5:**
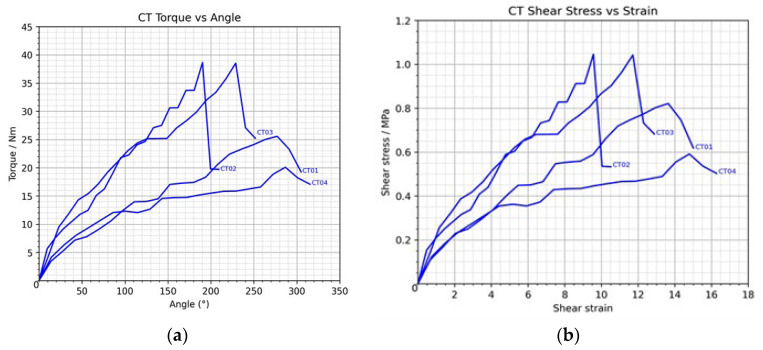
Graphs illustrating (**a**) the torque versus angular displacement curve and (**b**) the shear stress versus shear strain curve in the control group.

The mean maximum shear stress and shear strain were 0.88 ± 0.216 MPa and 12.43 ± 2.293, respectively, both increasing in parallel with torque and angular displacement. These findings indicate a direct proportional relationship between torque and shear stress, demonstrating consistent load-bearing and stress-distribution characteristics in the control group, as shown in Figure 5.

### 3.2. Damage Group Analysis

In the damage group (DM01–DM04), the mean peak torque was 10.30 ± 2.904 Nm, with DM01 demonstrating the highest value (13.38 Nm) and DM04 the lowest (6.90 Nm). The torque–angle curve exhibited a nonlinear pattern, characterized by a progressive increase between 100° and 250°, followed by a decline beyond 270°, reflecting tensile loading to the elbow’s structural limit. The mean maximum shear stress and shear strain were 0.21 ± 0.073 MPa and 9.28 ± 3.160, respectively, with DM01 presenting the highest values (0.31 MPa and 12.25). These findings indicate relatively superior mechanical performance in DM01 compared with other specimens in the group.

**Figure 6:**
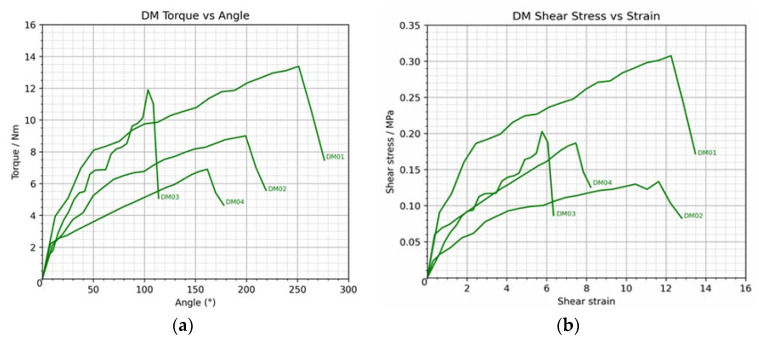
Graphs illustrating (**a**) the torque versus angular displacement curve and (**b**) the shear stress versus shear strain curve in the damage group.

Comparative analysis demonstrated significant differences between the control and damage groups across all mechanical variables. The control group exhibited a substantially greater mean peak torque (30.07 ± 9.368 Nm) than the damage group (10.30 ± 2.904 Nm), representing a reduction of approximately 66.5% following ligament compromise. Similarly, the mean maximum shear stress in the control group (0.88 ± 0.216 MPa) was more than fourfold higher than that of the damage group (0.21 ± 0.073 MPa), indicating markedly reduced post-damage shear loading capacity. The control group also demonstrated greater shear strain (12.43 ± 2.293 vs. 9.28 ± 3.160), suggesting a higher capacity for angular deformation in the intact elbow, as shown in Figure 6.

### 3.3. Repair Group Analysis

In the repaired group (RP01–RP04), the mean peak torque was 13.52 ± 1.58 Nm. RP04 had the highest value (15.56 Nm), and RP03 had the lowest (11.80 Nm). This shows that the mechanical performance was consistent after the lateral ulnar collateral ligament (LUCL) repair, with variations not exceeding 20%. The torque–angle relationship exhibited a nonlinear pattern, with torque increasing progressively up to approximately 120–140°, followed by a decline toward the end of testing, reflecting near-limit ligament elongation. The average maximum shear stress was 0.26 ± 0.070 MPa (range: 0.19–0.36 MPa), and the average maximum shear strain was 10.05 ± 3.505, which showed a steady increase as the torque increased.

**Figure 7:**
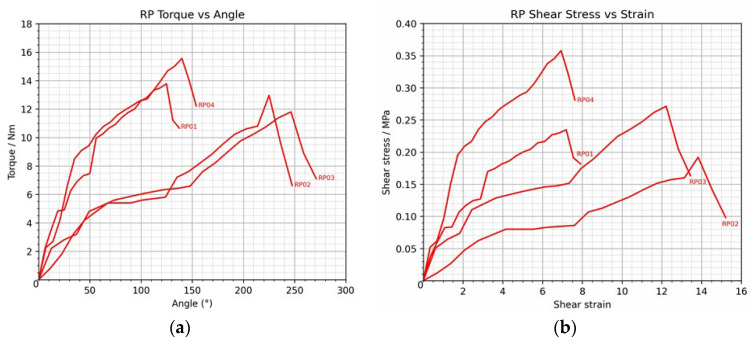
Graphs illustrating (**a**) the torque versus angular displacement curve and (**b**) the shear stress versus shear strain curve in the repair group.

Mechanical performance ranking across samples was RP04 > RP01 > RP02 > RP03 in both torque and shear stress, demonstrating stable torsional resistance and shear load distribution comparable to normal ligament function. RP04 exhibited the greatest restoration of biomechanical competence following repair, as shown in Figure 7.

### 3.4. Reconstruction Group Analysis

#### 3.4.1. Reconstruction RC-PL

Biomechanical testing of specimens reconstructed using the palmaris longus tendon (RC-PL01–RC-PL04) demonstrated a nonlinear torque–angle relationship, characterized by progressive torque increase during early to mid-range motion followed by a decline beyond 250–280°, consistent with ligament elongation nearing its structural limit. The mean peak torque was 16.62 ± 1.902 Nm, with RC-PL02 exhibiting the highest value (19.12 Nm) and RC-PL04 the lowest (14.50 Nm); peak torque occurred at 240–270°. The mean maximum shear stress and shear strain were 0.40 ± 0.064 MPa and 9.18 ± 3.531, respectively.

**Figure 8:**
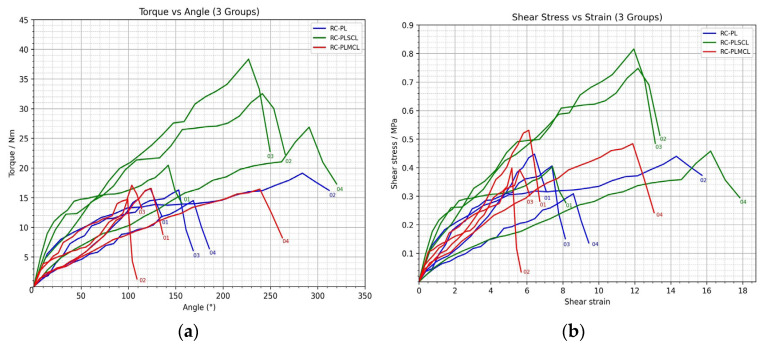
The graphs illustrate (**a**) the relationship between torque and angle as well as (**b**) shear stress and shear strain in all reconstruction groups.

Among the specimens, RC-PL02 demonstrated the highest mechanical performance in torque (19.12 Nm), shear stress (0.44 MPa), and shear strain (14.31), reflecting superior load-bearing capacity, whereas RC-PL04 showed lower torque but relatively high strain, indicating greater compliance. RC-PL01 and RC-PL03 had average performance, but RC-PL03 performed the best in the middle range of the angular phase. Overall, palmaris longus tendon reconstruction using the docking technique provided moderate-to-high torsional and shear resistance, with a direct proportional relationship between shear stress and shear strain, indicating consistent load distribution. RC-PL02 demonstrated the highest restoration of biomechanical function, suggesting effective mechanical recovery comparable to near-physiological elbow stability, as shown in Figure 8.

#### 3.4.2. Reconstruction RC-PLSCL

Biomechanical evaluation of synthetic ligaments constructed from the palmaris longus tendon combined with SCL-PHA co-polymer (RC-PLSCL01–RC-PLSCL04) demonstrated a nonlinear torque–angle response, characterized by a progressive torque increase from early to mid-range motion, followed by a decline near the material’s structural limit. Quantitative findings indicated notable inter-specimen variation in mechanical performance.

The mean peak torque was 29.55 ± 7.656 Nm, with a range from 20.46 Nm (RC-PLSCL01) to 38.34 Nm (RC-PLSCL03); peak torque occurred between 190° and 270°. The mean peak shear stress and shear strain were 0.61 ± 0.206 MPa and 11.94 ± 3.593, respectively. RC-PLSCL01 exhibited moderate mechanical capacity (peak torque, 20.46 Nm; shear stress, 0.40 MPa; shear strain, 7.42), whereas RC-PLSCL02 and RC-PLSCL03 demonstrated superior load-bearing performance (peak torque, 32.52–38.34 Nm; shear stress, 0.75–0.82 MPa; strain, 11.94–12.18). RC-PLSCL04 showed lower torque (26.88 Nm) but the greatest strain (16.21), indicating enhanced compliance and extensibility.

Overall, peak torque performance ranked RC-PLSCL01 < RC-PLSCL04 < RC-PLSCL02 < RC-PLSCL03, reflecting moderate-to-high torsional strength across specimens. Torque, shear stress, and shear strain increased proportionally in a nonlinear manner, indicating consistent load distribution and elastic–plastic deformation behavior. These findings suggest that hybrid palmaris longus–SCL-PHA co-polymer grafts provide substantial biomechanical strength with variable stiffness and extensibility profiles, with RC-PLSCL03 exhibiting the most robust mechanical characteristics, as shown in Figure 8.

#### 3.4.3. Reconstruction RC-PLMCL

Biomechanical testing of synthetic ligaments combining the palmaris longus tendon with MCL-PHA (RC-PLMCL01–RC-PLMCL04) demonstrated a nonlinear torque–angle profile, characterized by progressive torque elevation from early to mid-range motion followed by decline as the material approached its deformation limit. Mechanical performance varied among the specimens.

The mean peak torque was 16.20 ± 1.013 Nm, ranging from 14.74 Nm (RC-PLMCL02) to 17.08 Nm (RC-PLMCL03), with peak torque occurring between 120° and 180°. The mean peak shear stress and shear strain were 0.45 ± 0.067 MPa and 7.19 ± 3.155, respectively. RC-PLMCL01 exhibited moderate mechanical strength (16.54 Nm, 0.53 MPa, strain 6.11), whereas RC-PLMCL02 showed the lowest torque (14.74 Nm) but demonstrated acceptable toughness and baseline shear resistance. RC-PLMCL03 displayed the highest peak torque (17.08 Nm) with stable mid-range performance, while RC-PLMCL04 exhibited moderate torque (16.44 Nm) but the greatest strain (11.89), indicating high compliance and elongation capacity.

In general, the order of peak torque capacity was RC-PLMCL02 < RC-PLMCL04 < RC-PLMCL01 < RC-PLMCL03. This shows that all of the specimens had moderate to high mechanical competence. All samples exhibited nonlinear torque–angle behavior, with direct proportionality between shear stress and shear strain, indicating consistent load distribution and deformation response under torsional loading, as shown in Figure 8.

Finally, it should be noted that the raw biomechanical data underlying all analyses presented in this section, including individual torque–angle curves, shear stress, and shear strain values for each specimen across the control, damage, repair, and reconstruction groups, are provided in Supplementary Material S5. The inclusion of these unprocessed datasets allows for transparency, independent verification of the analytical results, and further secondary analyses, thereby strengthening the robustness and reproducibility of this study’s findings.

### 3.5. Comparison Among the Control, Damage, Repair, and Reconstruction Groups

A comparative analysis across the four groups revealed distinct mechanical differences. The control group demonstrated the highest performance, followed by the RC-PLSCL group, in which synthetic grafts reinforced with SCL-PHA co-polymer exhibited superior torque resistance and shear strength. The RC-PL group showed moderate biomechanical capacity, while the RC-PLMCL group, reinforced with MCL-PHA polymer, demonstrated comparable behavior with slightly lower torque yet preserved flexibility and toughness. The damage group presented the lowest mechanical performance, whereas the repair group, although still inferior to the control group in torque and shear parameters, exhibited consistent responses and a clear trend toward functional restoration.

**Figure 9:**
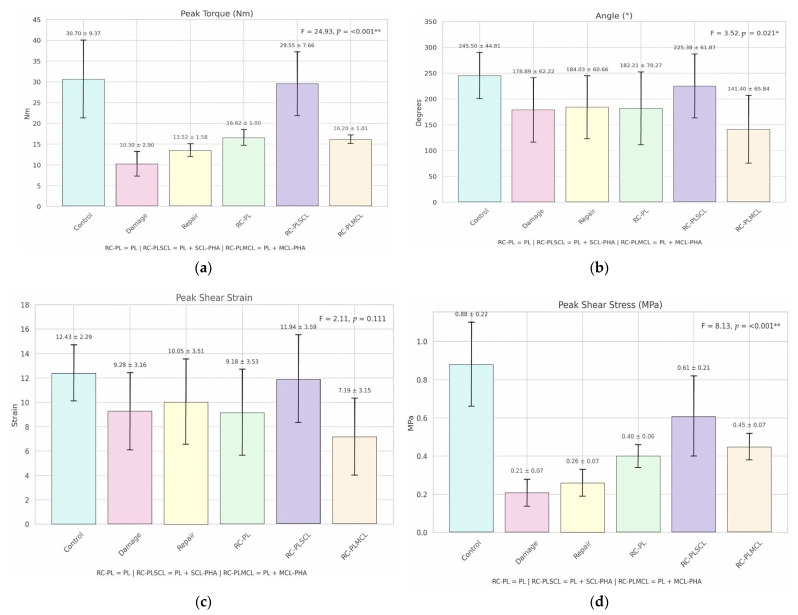
The graphs illustrate the mean values and standard deviations of (**a**) peak torque, (**b**) angle, (**c**) peak shear strain, and (**d**) peak shear stress across the experimental groups. Note: *p* < 0.05 indicates statistical significance; *p* < 0.001 indicates a highly significant difference.

The biomechanical properties of six subgroups—control, damage, repair, RC-PL, RC-PLSCL, and RC-PLMCL—were compared using one-way ANOVA. The results demonstrated statistically significant differences in peak torque among groups (F = 24.930, *p* < 0.001). A significant difference was also observed in the angle at peak torque (F = 3.520, *p* = 0.021), as well as in peak shear stress (F = 8.130, *p* < 0.001). In contrast, peak shear strain did not differ significantly across groups (F = 2.110, *p* = 0.111). Detailed results are presented in Table 1. Variables with statistically significant differences were further analyzed using post hoc multiple comparisons to identify specific intergroup differences, as summarized in Figure 9 and Table 2.

**Figure 10:**
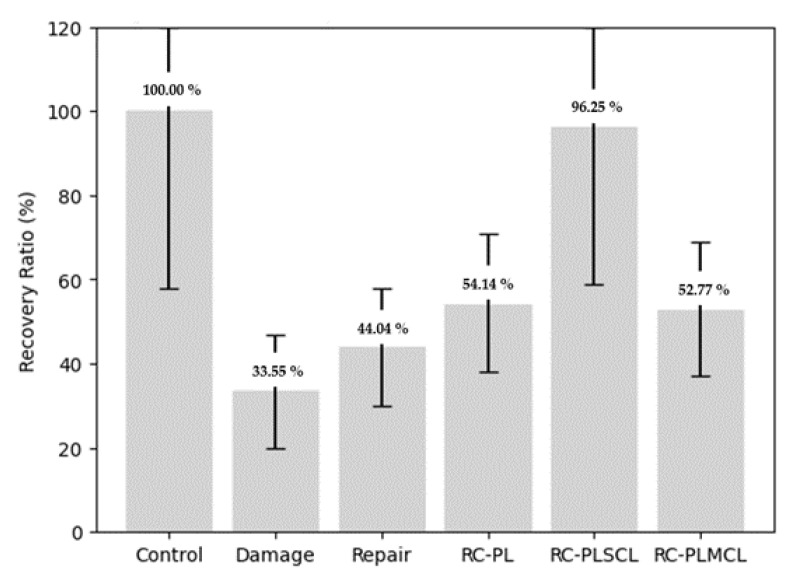
Comparison of recovery ratio (%) among the experimental groups, presented with 95% confidence intervals.

**Figure 11:**
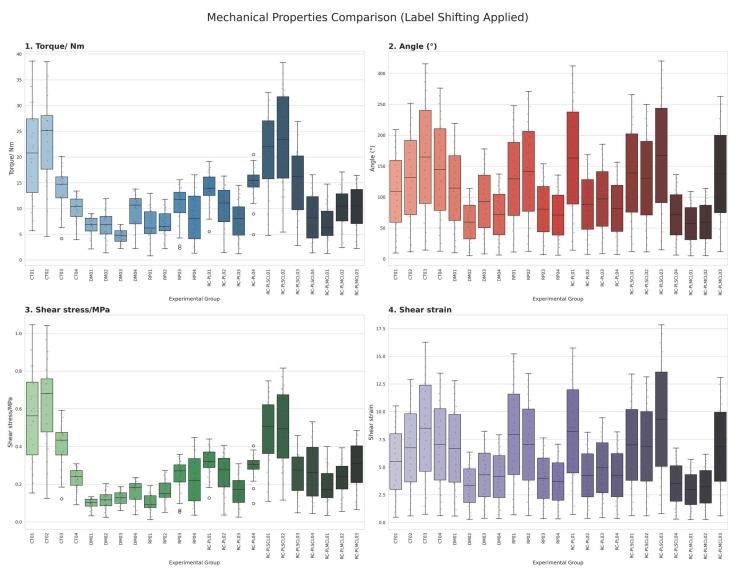
Boxplot visualization of biomechanical variability among experimental groups.

To further elucidate the biomechanical performance of the reconstructed ligaments, a recovery ratio was calculated from the peak torque values using the following formula: recovery ratio = (torque of the specimen/torque of the control group) × 100. This parameter represents the percentage of functional restoration achieved by each reconstructed ligament relative to the normal, intact ligament. The results showed that the RC-PLSCL group demonstrated the greatest functional recovery, achieving 96.25% of normal ligament strength. The RC-PL and RC-PLMCL groups exhibited moderate recovery ratios of 54.14% and 52.77%, respectively. In contrast, the damage and repair groups showed the lowest recovery ratios (33.55% and 44.04%), indicating limited biomechanical restoration (Figure 10, Table 1). In addition The distribution and variability of biomechanical parameters among the experimental groups are further illustrated in Figure 11 via the boxplot visualization technique.

### 3.6. Post Hoc Multiple Comparisons Were Performed to Evaluate Pairwise Differences

#### 3.6.1. Control Group

The control group demonstrated the highest peak torque (30.700 ± 9.368 Nm) compared with all other groups. Post hoc analysis revealed significant differences from the damage, repair, RC-PL, and RC-PLMCL groups (*p* < 0.05), but not from the RC-PLSCL group (*p* > 0.05). The mean angle at peak torque (245.500 ± 44.814°) was significantly greater than those of the damage, repair, RC-PL, and RC-PLMCL groups but did not differ from that of RC-PLSCL. Peak shear stress (0.880 ± 0.216 MPa) was significantly higher than that in nearly all groups. Peak shear strain (12.430 ± 2.293) showed no significant difference among groups (ANOVA: F = 2.110, *p* = 0.111).

#### 3.6.2. Damage Group

The damage group exhibited the lowest peak torque (10.300 ± 2.904 Nm), significantly lower than that in the control, repair, RC-PL, RC-PLSCL, and RC-PLMCL groups (*p* < 0.05). The mean angle (178.890 ± 62.218°) was significantly lower than those in the control, RC-PLSCL, and RC-PLMCL groups but did not differ from those in the repair and RC-PL groups. Peak shear stress (0.210 ± 0.073 MPa) was significantly lower than that in the control, RC-PL, RC-PLSCL, and RC-PLMCL groups.

#### 3.6.3. Repair Group

The repair group showed a peak torque of 13.520 ± 1.581 Nm, significantly different from all other groups. The mean angle (184.030 ± 60.655°) was significantly lower than those in the control, RC-PLSCL, and RC-PLMCL groups but did not differ from those in the damage and RC-PL groups. Peak shear stress (0.260 ± 0.070 MPa) differed significantly from that in most groups. Peak shear strain (10.050 ± 3.505) did not significantly differ among groups.

#### 3.6.4. RC-PL Group

The RC-PL group exhibited a peak torque of 16.620 ± 1.902 Nm, significantly different from that in the control, damage, repair, RC-PLSCL, and RC-PLMCL groups, although some pairwise differences were small. The mean angle (182.210 ± 70.270°) differed significantly from those in the control group and selected pairs. Peak shear stress (0.400 ± 0.064 MPa) was significantly different from that in most groups, whereas peak shear strain (9.180 ± 3.531) showed no significant difference.

#### 3.6.5. RC-PLSCL Group

The RC-PLSCL group demonstrated a peak torque of 29.550 ± 7.656 Nm, comparable to the control group and significantly different from most other groups. The mean angle (225.380 ± 61.873°) differed significantly from those in the damage, repair, RC-PL, and RC-PLMCL groups, but not from that in the control group. Peak shear stress (0.610 ± 0.206 MPa) was significantly different from that in several groups, while peak shear strain (11.940 ± 3.593) showed no significant difference.

#### 3.6.6. RC-PLMCL Group

**Figure 12:**
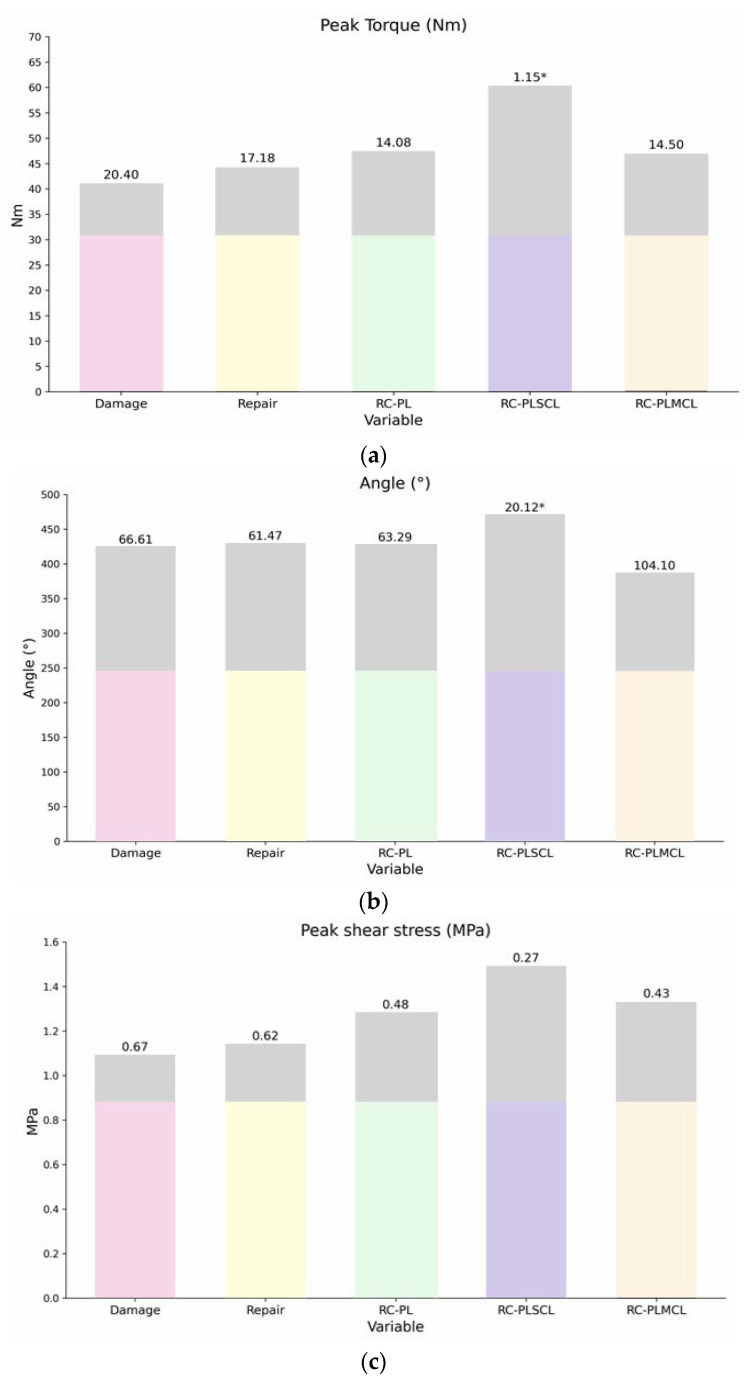
Biomechanical comparison among experimental groups: (**a**) peak torque (Nm); (**b**) angular displacement at peak torque (°); (**c**) peak shear stress (MPa). Bars represent mean values, and an asterisk (*) indicates a statistically significant difference compared with the control group (*p* < 0.05). The gray bars represent the mean difference (Mean Diff).

The RC-PLMCL group had a peak torque of 16.200 ± 1.013 Nm, significantly different from that in the control, damage, repair, and RC-PLSCL groups, but not from that in the RC-PL group. The mean angle (141.400 ± 65.842°) was the lowest among all groups and differed significantly from those in multiple groups. Peak shear stress (0.450 ± 0.067 MPa) differed significantly from that in several groups, whereas peak shear strain (7.190 ± 3.155) showed no significant difference. Detailed results are presented in Figure 12.

When the reconstructed groups were compared with the control group, the differences in peak torque, angular displacement, and shear properties reflected the underlying mechanical behavior and load-transfer capabilities of each material. The markedly higher peak torque observed in the RC-PLSCL group, which approached the performance of the native ligament, suggests that SCL-PHA co-polymer possesses superior toughness and load-bearing capacity, enabling more effective transmission of tensile forces across the reconstructed ligament. In contrast, the RC-PL and RC-PLMCL groups demonstrated only moderate torque recovery, indicating limited energy absorption and reduced resistance to deformation under load—properties consistent with the lower stiffness and increased ductility typically associated with MCL-PHA. The damage and repair groups exhibited the weakest biomechanical response, characterized by low peak torque and shear stress, reflecting compromised structural integrity and insufficient ligament stability. Together, these findings highlight that variations in material properties—such as molecular structure, crystallinity, and fiber cohesion—directly influence the ligament’s ability to withstand torsional and shear loading, explaining the observed differences relative to the intact control.

## 4. Discussion

In this study, the biomechanical characteristics of ligaments were compared across normal, injured, repaired, and reconstructed conditions to identify which approach most effectively restores native mechanical behavior. As expected, normal ligaments served as the reference standard, displaying the highest peak torque, angular displacement, and shear resistance. Injured ligaments showed marked reductions in mechanical capacity, reflecting the disruption of collagen organization and compromised load-bearing ability. Although repaired ligaments demonstrated partial recovery, their mechanical performance remained inferior to that of native tissue. When contextualized against physiological loading demands—where daily forearm rotation typically generates 4–5 Nm and high-demand activities may reach 10–15 Nm—the peak torque of the RC-PLSCL reconstructed group (~29.55 Nm) substantially exceeded functional requirements, indicating that this construct possesses ample mechanical robustness to withstand both routine and elevated rotational forces. These findings support the conclusion that the reconstructed ligament is mechanically compatible with real-world elbow-loading conditions.

Failure patterns provided further insight into structural performance. Injured and conventionally repaired ligaments predominantly failed near the proximal insertion site, consistent with stress concentration and disrupted collagen alignment. In contrast, constructs reinforced with SCL-PHA co-polymer failed at the graft–bone tunnel interface or along the augmentation strand rather than through the mid-substance. This shift suggests that SCL-PHA co-polymer reinforcement enhances load redistribution and reduces focal stress, thereby improving resistance to rotational deformation. Reconstruction using the docking technique—particularly with a palmaris longus autograft combined with SCL-PHA co-polymer augmentation—yielded torque and shear resistance most comparable to the native ligament. These reinforced constructs also preserved physiological elongation behavior and demonstrated minimal rotational deformation, indicating excellent mechanical compatibility between the biological graft and the synthetic augment. This pattern aligns with previous evidence identifying polyhydroxyalkanoates (PHAs) as promising biomaterials owing to their load-bearing potential, biodegradability, and tunable mechanical properties suitable for mimicking human soft tissues [20,21]. Although the RC-PLMCL construct did not match the strength of the SCL-PHA co-polymer-reinforced group, it exhibited favorable elasticity and toughness—features that may mitigate micro-failure by permitting controlled deformation while maintaining ligament kinematics. Beyond their favorable early-phase mechanical performance, PHAs offer distinct advantages over permanent synthetic augmentation materials (e.g., PET- or UHMWPE-based constructs) commonly used in ligament reconstruction. Unlike permanent synthetics, which remain indefinitely in situ and may be predisposed to chronic foreign-body reactions, stress shielding, or late mechanical fatigue, biodegradable PHA-based materials are designed to undergo gradual resorption and replacement by neotissue. This degradation-mediated load transfer may promote more physiological remodeling and reduce long-term implant-related complications, while still providing sufficient initial mechanical support during the critical healing period.

In terms of long-term performance, the in vivo degradation behavior of PHA-based materials represents a critical determinant of post-remodeling mechanical stability. Previous studies have demonstrated that the degradation rate of PHAs is strongly influenced by polymer chain length, crystallinity, and microstructural organization. Short-chain-length PHA co-polymers such as PHBHHx typically exhibit slower degradation kinetics due to their relatively higher crystallinity, enabling mechanical load-bearing to be maintained for several months to over one year in vivo, depending on composition and implantation site [20,21,22]. In contrast, medium-chain-length PHAs (MCL-PHAs) possess lower crystallinity and greater chain mobility, resulting in faster degradation profiles and increased elasticity but potentially reduced long-term mechanical retention [10,12]. Within the context of ligament reconstruction, this differential degradation behavior has important biomechanical implications. Premature polymer degradation may compromise structural stability before sufficient neotissue formation occurs, whereas excessively slow degradation may induce stress shielding and impede physiological load transfer to the regenerating ligament. The present biomechanical findings—demonstrating superior early-phase mechanical performance in SCL-PHA-reinforced constructs—suggest that the degradation profile of SCL-PHA co-polymers may be well aligned with the temporal demands of ligament healing, providing initial stability while allowing gradual load transfer during tissue remodeling. Nevertheless, dedicated long-term in vivo investigations are required to elucidate the coupled degradation–remodeling kinetics and to define the optimal balance between polymer resorption and biological ligament regeneration [23,24].

Clinically, these results are consistent with established anatomical and biomechanical evidence demonstrating the critical role of the LUCL in preserving elbow stability, particularly at flexion angles exceeding 60 degrees [25]. The findings also reflect current trends in biomaterial augmentation aimed at increasing joint stiffness and preventing excessive motion during the early postoperative period [22,23]. The mechanical behavior of the SCL-PHA co-polymer-reinforced constructs observed in this study aligns well with these evolving concepts of early-phase ligament reinforcement.

Furthermore, the biomechanical performance demonstrated in this study shows strong concordance with prior cadaveric evidence concerning ligament reconstruction and augmentation. In agreement with Dugas et al. [26], who reported that internal brace augmentation significantly enhances valgus stability, torsional resistance, and early construct stiffness in UCL repair, the SCL-PHA co-polymer-reinforced constructs here similarly exhibited superior torque resistance, reduced abnormal elongation, and improved early-phase mechanical stability. The restoration of posterolateral rotatory control observed in the RC-PLSCL group also parallels the improvements described by Hackl et al. [27], who demonstrated that LUCL reconstruction effectively restores stability against posterolateral rotatory instability. In addition, the reinforced constructs exhibited high torque resistance, enhanced rotational control, and minimal deformation, findings consistent with Vopat et al. [28], who showed that reconstruction techniques emphasizing precise isometric fixation—such as the docking method—yield superior stiffness and valgus stability compared with alternative approaches. The shifted failure pattern toward the graft–bone interface also mirrors the effect reported by Narvaez et al. [22], who demonstrated that suture augmentation strengthens mid-substance mechanics, reduces deformation under cyclic loading, and alters failure modes by enhancing load sharing. Collectively, these concordant findings indicate that SCL-PHA co-polymer augmentation substantially improves graft mechanical performance, optimizes load distribution, and reduces vulnerability to early mechanical failure, particularly when integrated with anatomically accurate docking-technique reconstruction that closely replicates native ligament biomechanics.

Despite these promising findings, several limitations should be acknowledged. The use of embalmed cadaveric specimens may alter tissue elasticity and viscoelastic behavior as a result of formalin-induced protein cross-linking, potentially leading to overestimation of tissue stiffness when compared with in vivo conditions. Consequently, the absolute mechanical values reported in this study should be interpreted as indicative of relative biomechanical trends rather than precise physiological thresholds. Nevertheless, all specimens were obtained from the same source, preserved using an identical embalming protocol, and tested under standardized experimental conditions, thereby permitting valid comparative analyses across the control, damage, repair, and reconstruction groups. In addition, the relatively small sample size in each experimental group (*n* = 4) may limit statistical power and increase the risk of type II error. This constraint was primarily related to the availability and ethical use of cadaveric specimens and is consistent with prior exploratory cadaveric biomechanical investigations. Despite this limitation, the consistency of observed effect directions across experimental conditions supports the internal validity of the comparative findings. Furthermore, this investigation was limited to ex vivo biomechanical evaluation and did not include in vivo validation or biological assessments such as histological or immunological analyses. As a result, tissue integration, host inflammatory response, and graft–material interface remodeling could not be directly assessed, which restricts conclusions regarding biological incorporation and long-term biocompatibility. In addition, although polyhydroxyalkanoates are biodegradable and designed to be gradually replaced by neotissue, the present cadaveric model does not allow the evaluation of in vivo degradation kinetics or their potential impact on long-term construct stability following ligament remodeling. To further enhance translational relevance, future investigations should consider the use of fresh-frozen cadaveric specimens, larger sample sizes, cyclic or fatigue loading protocols, and well-designed in vivo models incorporating histological, immunological, and longitudinal assessments to better characterize the viscoelastic response, degradation–remodeling behavior, and clinical durability of PHA-augmented ligament reconstruction strategies.

## 5. Conclusions

Biopolymer-reinforced ligament reconstruction, particularly using SCL-PHA co-polymer, demonstrated strong potential to restore the mechanical stability and physiological deformation behavior of injured ligaments to a level closely approximating that of native tissue. This strategy represents a promising pathway for future clinical application in patients requiring high mechanical durability and resistance to repetitive loading, such as athletes and individuals performing demanding upper-extremity activities.

## Figures and Tables

**Table 1:** Comparison of means and standard deviations among the control, damage, repair, and reconstruction groups using one-way ANOVA (*n* = 24).

Variable	Control (*n* = 4)	Damage (*n* = 4)	Repair (*n* = 4)	RC-PL (*n* = 4)	RC-PLSCL (*n* = 4)	RC-PLMCL (*n* = 4)	F-Value	*p*-Value
Peak torque (Nm)	30.70 ± 9.37	10.30 ± 2.90	13.52 ± 1.58	16.62 ± 1.90	29.55 ± 7.66	16.20 ± 1.01	24.930	<0.001 **
Recovery ratio (%)	100.00	33.55	44.04	54.14	96.25	52.77	-	-
Angle (°)	245.50 ± 44.81	178.89 ± 62.22	184.03 ± 60.66	182.21 ± 70.27	225.38 ± 61.87	141.40 ± 65.84	3.520	0.021 *
Peak shear stress (MPa)	0.88 ± 0.22	0.21 ± 0.07	0.26 ± 0.07	0.40 ± 0.06	0.61 ± 0.21	0.45 ± 0.07	8.130	<0.001 **
Peak shear strain	12.43 ± 2.29	9.28 ± 3.16	10.05 ± 3.51	9.18 ± 3.53	11.94 ± 3.59	7.19 ± 3.15	2.110	0.111

Note: * *p* < 0.05 indicates statistical significance; ** *p* < 0.001 indicates a highly significant difference (one-way ANOVA).

**Table 2:** Post hoc multiple comparisons for pairwise group differences.

Variable	Group 1	Group 2	Mean Diff	SE	*t*-Stat	*p*-Value	Significant
Peak torque (Nm)	Control	Damage	20.40	2.002	10.19	<0.001	Yes
	Control	Repair	17.18	1.939	8.86	<0.001	Yes
	Control	RC-PL	14.08	1.951	7.22	<0.001	Yes
	Control	RC-PLSCL	1.15	2.470	0.47	0.639	No
	Control	RC-PLMCL	14.50	1.923	7.54	<0.001	Yes
Angle (°)	Control	Damage	66.61	15.652	4.26	0.002	Yes
	Control	Repair	61.47	15.394	3.99	0.003	Yes
	Control	RC-PL	63.29	17.012	3.72	0.004	Yes
	Control	RC-PLSCL	20.12	15.595	1.29	0.215	No
	Control	RC-PLMCL	104.10	16.258	6.40	<0.001	Yes
Peak shear stress (MPa)	Control	Damage	0.67	0.047	14.40	<0.001	Yes
	Control	Repair	0.62	0.046	13.38	<0.001	Yes
	Control	RC-PL	0.48	0.046	10.44	<0.001	Yes
	Control	RC-PLSCL	0.27	0.061	4.43	0.007	Yes
	Control	RC-PLMCL	0.43	0.046	9.31	<0.001	Yes

## Data Availability

Dataset available on request from the authors.

## Supplementary Materials

The following supporting information can be downloaded at: https://www.mdpi.com/article/10.3390/polym18040514/s1. The procedure videos in the repair and reconstruction groups: S1, S2, S3, S4, and S5. S1: Muscle Dissection and LUCL Repair; S2: Docking Technique-Based Reconstruction Using the PL; S3: Docking Technique-Based Reconstruction Using the PL with PHA; S4: Testing was performed using a LLOYD testing machine; and S5: Biomechanical test data.
